# Genotypes and phenotypes of patients with Lafora disease living in Germany

**DOI:** 10.1186/s42466-019-0040-2

**Published:** 2019-11-12

**Authors:** David Brenner, Tobias Baumgartner, Sarah von Spiczak, Jan Lewerenz, Roger Weis, Anja Grimmer, Petra Gaspirova, Claudia D. Wurster, Wolfram S. Kunz, Jan Wagner, Berge A. Minassian, Christian E. Elger, Albert C. Ludolph, Saskia Biskup, Dennis Döcker

**Affiliations:** 1 Department of Neurology, University of Ulm, Ulm, Germany; 2 Department of Epileptology, University of Bonn Hospital, Bonn, Germany; 3 DRK-Northern German Epilepsy Center for Children and Adolescents, Schwentinental, Raisdorf, Germany; 4 Neuropediatric Center, Rheinhessen-Fachklinik Mainz, Mainz, Germany; 5 Epilepsie-Zentrum Berlin-Brandenburg, Berlin, Germany; 6 Klinikum St. Marien Amberg, Amberg, Germany; 7 Institute of Experimental Epileptology and Cognition Research, University Bonn, Bonn, Germany; 8 Department of Pediatrics, University of Texas Southwestern, Dallas, USA; 9 Center for Genomics and Transcriptomics (CeGaT) GmbH and Practice for Human Genetics, Tübingen, Germany

## Abstract

**Background:**

Lafora progressive myoclonus epilepsy (Lafora disease) is a rare, usually childhood-onset, fatal neurodegenerative disease caused by biallelic mutations in *EPM2A* (Laforin) or *EPM2B* (*NHLRC1*; Malin)*.* The epidemiology of Lafora disease in Germany is largely unknown. The objective of this retrospective case series is to characterize the genotypes and phenotypes of patients with Lafora disease living in Germany.

**Methods:**

The patients described in this case series initially had the suspected clinical diagnosis of Lafora disease, or unclassified progressive myoclonus epilepsy. Molecular genetic diagnostics including next generation sequencing-based diagnostic panel analysis or whole exome sequencing was performed.

**Results:**

The parents of four out of the 11 patients are nonconsanguineous and of German origin while the other patients had consanguineous parents. Various variants were found in *EPM2A* (six patients) and in *EPM2B* (five patients). Eight variants have not been reported in the literature so far. The patients bearing novel variants had typical disease onset during adolescence and show classical disease courses.

**Conclusions:**

This is the first larger case series of Lafora patients in Germany. Our data enable an approximation of the prevalence of manifest Lafora disease in Germany to 1,69 per 10 million people. Broader application of gene panel or whole-exome diagnostics helps clarifying unclassified progressive myoclonus epilepsy and establish an early diagnosis, which will be even more important as causal therapy approaches have been developed and are soon to be tested in a phase I study.

**Electronic supplementary material:**

The online version of this article (10.1186/s42466-019-0040-2) contains supplementary material, which is available to authorized users.

## Introduction

Lafora disease (LD; EPM2A/B; OMIM #254780) is a severe form of progressive myoclonus epilepsy inherited in an autosomal recessive mode and caused by biallelic mutations in *EPM2A* (Laforin) or *EPM2B* (*NHLRC1;* Malin) [1, 2]. Pathogenic homozygous or compound heterozygous mutations in both genes cause cytoplasmatic precipitation, aggregation and accumulation of neurotoxic poorly branched and insoluble glycogen forming polyglucosan inclusions (so-called Lafora bodies) leading to progressive neurodegeneration. In most cases, the disease starts with epileptic seizures in late childhood or adolescence. Apart from multiple types of seizures (tonic-clonic, myoclonic, absence, atonic, or visual), LD patients develop progressive cerebellar ataxia, dysarthria, dementia and neuropsychiatric symptoms. LD usually leads to death within 10 years after symptom onset. The prevalence is below 1–9 in 10^6^, depending on the population and the frequency of consanguinity [3]. The epidemiology as well as the genotypic and phenotypic spectrum of LD in Germany is largely unknown. At present, therapy is merely symptomatic and palliative. Recent preclinic studies have shown that downregulation of glycogen synthesis prevents LD in mice [4]. Antisense oligonucleotides (ASO) targeting the glycogen synthase mRNA significantly reduce Lafora body load in LD mice (unpublished observation). Consequently, an ASO targeting the human glycogen synthase mRNA is being developed towards an upcoming clinical trial. A two-year natural history study prerequisite for this therapy trial is currently ongoing (NCT03876522).

Here, we describe the genotypes and phenotypes of 11 index patients with LD living in Germany.

## Methods

### DNA analysis

Molecular diagnostics was performed as part of the clinical follow-up in all cases. DNA was extracted from EDTA blood samples according to standard procedures. Next generation sequencing (NGS)-based diagnostic panel analysis or whole exome sequencing was performed in 11 index patients with suspected clinical diagnosis of Lafora disease, or unclassified progressive myoclonus epilepsy. Annotation of reported variants is based on human reference genome *Homo sapiens* GRCh37 (hg19). All patients or parents, respectively, gave their consent to publication of their cases.

## Results

Four out of the 11 patients are of German origin. Variants in *EPM2A* or *EPM2B* were detected by NGS in all 11 index patients with suspected LD or unclassified progressive myoclonus epilepsy. Variants of various types were detected in *EPM2A* in six patients (three of them of German origin) and in *EPM2B* in five patients (one of them of German origin; see Table 1). Five *EPM2A* variants (c.259A > G, c.290 T > G, c.759delinsCATGCA, c.836G > T, c.917A > T) and three *EPM2B* variants (c.385C > T, c.583del, and c.730delG) have not been reported in the LD literature or in the Lafora gene mutation database (http://projects.tcag.ca/lafora/) before [5]. The mean age of disease onset is 13,7 ± 0,6 years (mean ± SEM) in the patients bearing *EPM2A* variants and 14,6 ± 2,3 years in those carrying *EPM2B* variants. The cumulative mean age of onset of this cohort is 14,1 ± 1,0 years (range: 8–21 years).

**Table 1:** Genotypes of LD patients living in Germany

P. nr.	Variant			Alleles	Ref.
	*EPM2A*				
	NC_000006.11:	NM_005670.3:			
1^a^	g.146056376 T > C	c.259A > G	p.(Lys87Glu)	Homo.	
2^a^	g.[146056360_146056366del]; [145,948,631 T > A]	c.[269_275del];[917A > T]	p.[(Lys90Serfs*35);(Asp306Val)]	Comp. het.	[6]
3^a^	g.146056345A > C	c.290 T > G	p.(Leu97Arg)	Homo.	
4	g.146007412G > A	c.322C > T	p.(Arg108Cys)	Homo.	[1]
5^a^	g.145948789delinsTGCATG	c.759delinsCATGCA	p.(Ala254Metfs*33)	Homo.	
6^a^	g.145948712C > A	c.836G > T	p.(Gly279Val)	Homo.	
	*EPM2B*				
	NC_000006.11:	NM_198586.2:			
7^a^	g.18122453G > A	c.385C > T	p.(Pro129Ser)	Homo.	
8, 9	g.18122402C > T	c.436G > A	p.Asp146Asn	Homo.	[2, 7]
10^a^	g.[18122402C > T]; [18122108delC]	c.[436G > A];[730delG]	p.[(Asp146Asn);(Val244Serfs*51)]	Comp. het.	[2, 7]
11^a^	g.18122255del	c.583del	p.(Asp195Ilefs*37)	Homo.	

Patients with novel variants are indicated by a superscripted letter (^a^). Annotation based on human reference genome *Homo sapiens* GRCh37 (hg19)

In the following paragraphs, we shortly outline the disease courses of the patients bearing novel variants (also see Table 2). The anticonvulsants valproate (11/12), perampanel (8/12), levetiracetam/brivaracetam (7/12), and clobazam (6/12) were the most used ones in our cohort indicating a good of effectiveness of these drugs in LD (see Additional file 1: Table S1).

**Table 2:** Disease courses of LD patients living in Germany

Patient number	Nationality	Gender	Consanguinity	Variant	Age of onset				Current age	Current disease stage	Special features
					Tonic-clonic seizure	Myoclonus	Ataxia	Cognitive decline			
				*EMP2A*							
1^a^	Tur	F	yes	c.259A > G (homo.)	14	15	15	15	18	4	Gelastic seizures
2^a^	Ger	F	no	c.[269_275del];[917A > T]	17	15	17	16	18	3–4	Congenital hypothyroidism, polyneuropathy
3^a^	Ger	F	no	c.290 T > G (homo.)	14	15	17	17	25	3	Psychogenic seizures
4a	Leb	F	yes	c.322C > T (homo.)	12	n/a	n/a	n/a	18	4	Vision loss
4b	Leb	M	yes	c.322C > T (homo.)	11	n/a	n/a	< 16	16	1–2	
5^a^	Rus	M	yes	c.759delinsCATGCA	14	14	17	15	21	3	Aggressiveness
6^a^	Ger	M	no	c.836G > T (homo.)	18	16	19	20	24	3	
				*EMP2B*							
7^a^	Syr	F	yes	c.385C > T (homo.)	11	15	15	15	19	4	Optic hallucinations, hypothyroidism, hepatomegaly
8	Tur	M	yes	c.436G > A (homo.)	17	17	23	26	30	3–4	Diabetes mellitus with coma and cataract, hypothyroidism, arterial hypertension
9	Tur	F	yes	c.436G > A (homo.)	23	21	–	23	25	2	
10^a^	Ger	F	no	c.[436G > A];[730delG]	16	17	–	–	23	1	
11^a^	Ira	M	yes	c.583del (homo.)	8	13	–	8	13	1–2	Psychogenic seizures

Patients with novel variants are indicated by a superscripted letter (^a^). Patients 4 a and b are siblings. *Abbreviations*: *Ger* German, *Ira* Iraqi, *Leb* Lebanese, *Rus* Russian, *Syr* Syrian, *Tur* Turkish. Disease stage score was assessed according to Ferlazzo et al. [8]: (1) mild cognitive and motor impairment, preserved daily living activities, and social interaction; (2) moderate mental decline, limitations in motor activities, and limited social interaction; (3) severe mental and motor impairment, needing help in walking and regular assistance in daily living activity, and poor social interaction; (4) patient wheelchair-bound or bedridden, and no significant daily living activities or social interaction. Consanguinity or non-consanguinity status was based on family history

### Case vignettes of index patients with novel variants (Table 2)

**Patient nr. 1** is a homozygous carrier of the *EPM2A* variant c.259A > G. Her parents are of Turkish origin and are consanguineous. Retrospectively, the first symptom were recurrent episodes of explosive headache at the age of 12 years. Next, myoclonic and atonic seizures with consecutive falls developed. Aged 15 years, she developed tonic-clonic, visual, gelastic and absence seizures and (negative) myoclonus aggravated. Shortly later, she also developed severe ataxia and progressive cognitive decline. At the age of 19 years, the patient is wheelchair-bound and exhibits severe dysarthria as well as dysphagia requiring percutaneous endoscopic gastrostomy (PEG).

**Patient nr. 2** is compound heterozygous for the known variant c.269_275del and the novel variant c.917A > T in *EPM2A*. Her parents are German and nonconsanguineous. She developed myoclonic seizures as first disease symptom at the age of 15 years. However, an EEG at the age of 6 years, performed due to headaches showed posterior slow waves, which were interpreted as a variant of the basic rhythm at that time. Since the beginning of the epilepsy, the young woman lost major cognitive and motor functions and suffers from ataxia. She developed polyneuropathy of the lower extremities. About 3 years later, she can walk only few steps with lots of support and needs help with all activities of daily living.

**Patient nr. 3** is a homozygous carrier of the *EPM2A* variant c.290 T > G. She is of German origin and her parents are considered nonconsanguineous based on family history. She had onset of tonic-clonic seizures at the age of 14 years. Shortly later, she developed myoclonic and absence seizures, ataxia and cognitive decline. She exhibits a slow progression and is still able to walk and speak 12 years after onset of symptoms.

**Patient nr. 5** is homozygous for the *EPM2A* variant c.759delinsCATGCA. His parents are Russian and are consanguineous. He developed tonic-clonic seizures at the age of 15 years. The patient showed striking social difficulties and increased aggressiveness. After the age of 17 years, psychomotor skills reduced, and the patient developed tremor, ataxia and progressive cognitive decline. At the age of 20, he has lost many everyday skills and suffers from frequent seizures, while refusing medication. Due to severe dysphagia a PEG was implanted. At the age of 23 years he is wheelchair-bound and shows severe dysarthria.

**Patient nr. 6** carries the homozygous c.836G > T variantin *EPM2A*. He is of German origin and his parents are considered nonconsanguineous based on family history. Myoclonic seizures started at the age of 16. About the same time, visual auras occurred followed by tonic-clonic and absence seizures about 1 year later. Thereafter, he developed mild ataxia and a cognitive decline. At the age of 24, daily drop attacks and myoclonic seizures were the main problem of the patient. His sister carried the same homozygous mutation in *EPM2A*, but her course of the disease was more severe and she died at the age of 24.

**Patient nr. 7** carries the homozygous c.385C > T variant in *EPM2B*. She is of Syrian origin and her parents are consanguineous. She suffered the first and second tonic-clonic seizure at the age of 11 and 13 years. Thereafter, the frequency of seizures increased and since the age of 15, she has been suffering from intermittent myoclonic seizures and progressive dysarthria, ataxia, optic hallucinations and dementia. At the current age of 19 years, the patient is wheelchair-bound and suffers from severe dysphagia requiring PEG.

**Patient nr. 10** is compound heterozygous for the known variant c.436G > A and the novel variant c.730delG in *EPM2B*. She is German and her parents are nonconsanguineous. She first developed tonic-clonic and absence seizures at the age of 16. About 1 year later, myoclonic seizures started. A juvenile myoclonic epilepsy was suspected. After initiating a therapy with Valproate, tonic-clonic seizures were well under control for about 4 years. Meanwhile, myoclonic seizures accelerated under different medications and the EEG worsened. At the age of 23, the patient’s cognitive skills are still preserved, and he does not suffer from ataxia.

**Patient nr. 11** is homozygous for the c.583del variant in *EPM2B*. He is Iraqi and his parents are consanguineous. His epilepsy started at the age of 9 years with generalized tonic-clonic seizures. Later, he developed myoclonic seizures (especially negative myoclonus), psychogenic seizures and cognitive decline. His motor abilities are relatively well preserved (now aged 14).

## Discussion

This is the first larger case series of Lafora patients in Germany. Consistent with consanguinity being uncommon in Germany and the very rare prevalence of this recessive disease, only four out of the 11 patients are of German origin. In our cohort of LD patients, variants in *EPM2A* and *EPM2B* show a similar frequency. We report eight novel variants in *EPM2A* and *EPM2B*. Six novel variants are located maximally three nucleotides away from aforedescribed pathogenic variants [5]. While the parents of the seven Non-German patients were consanguineous, those of the four German patients were nonconsanguineous. Although not verifiable by family history, the fact that two out of the four German patients bear homozygous variants should indicate that there may be a common ancestor. The patients bearing novel variants in *EPM2A* or *EPM2B* had typical disease onset during adolescence and developed characteristic symptoms including multiple types of seizures, ataxia, dysarthria and dysphagia, as well as cognitive decline during the disease course. The cumulative mean age of onset of this LD patient cohort of 14 ± 1 years coincides with the reported peak age of onset [3]. Consistent with previous reports [6], the three patients bearing the known *EPM2B* c.436G > A variant (patients nr. 8–10) had a late onset of symptoms and show a comparatively slow disease progression. Remarkably, the 35-year old sister of patient nr. 8 who is also homozygous for the c.436G > A variant (the genetic result was confirmed twice by independent laboratories) is asymptomatic to date. Noteworthy, patient nr. 8 bearing the *EPM2B* c.436G > A variant developed severe diabetes mellitus with diabetic coma and cataract, a finding that recently has also been described in a LD patient with an *EPM2B* c.386C > A variant [9]. Further, patient nr. 2 bearing the c.[269_275del];[917A > T] variants in *EPM2A* developed a polyneuropathy of the lower limbs. Although a causal link remains speculative, polyglucosan formation in axons indeed causes severe axonopathy in patients with adult polyglucosan body disease [10]. Based on the data of this report and the knowledge abou two additional LD patients not included here the estimated prevalence of manifest LD in Germany is 14 per 83.019.000 [11] or 1,69 per 10 million people, respectively. Including the pre-symptomatic mutation carriers known to us the prevalence raises to 2,05 per per 10 million people.

## Conclusions

This is the first larger case series of Lafora patients in Germany. Our data enable an approximation of the prevalence of manifest Lafora disease in Germany to 1,69 per 10 million people. Early genetic testing is central when suspecting this condition given that causal therapy approaches have been developed and are soon to be tested in a phase I study. Broader application of gene panel or whole-exome diagnostics helps clarifying unclassified PME and establish an early diagnosis.

## Additional file

Additional file 1: **Table S1.** Anticonvulsive pharmacotherapy of LD patients living in Germany. (DOCX 16 kb)

## Data Availability

The datasets used and analysed during the current study are available from the corresponding author on reasonable request.

## Abbreviations

ASO: Antisense oligonucleotide
LD: Lafora disease
NGS: Next generation sequencing
PEG: Percutaneous endoscopic gastrostomy
PME: Progressive myoclonus epilepsy
