# Supplementary material for: Genotypes and phenotypes of patients with Lafora disease living in Germany
Source: Neurol Res Pract. 2019 Nov 12;1:34. doi: 10.1186/s42466-019-0040-2 (PMC7316188; doi:10.1186/s42466-019-0040-2)
Supplement: Supplementary file 1 — Table S1. Anticonvulsive pharmacotherapy of LD patients living in Germany. (DOCX 16 kb) [file 42466_2019_40_MOESM1_ESM.docx]

Additional file 1: **Table S1** Anticonvulsive pharmacotherapy of LD patients living in Germany.

| **Patient number** | **Nationality** | **Gender** | **Variant** | **Current medication** |
| --- | --- | --- | --- | --- |
|  |  |  | ***EMP2A*** |  |
| 1* | Tur | F | c.259A>G (homo.) | Perampanel, valproate und clobazam |
| 2* | Ger | F | c.[269_275del];[917A>T] | Valproate, clobazam, stiripentol, perampanel |
| 3* | Ger | F | c.290T>G (homo.) | Levetiracetam, piracetam, perampanel, clobazam, valproate |
| 4a | Leb | F | c.322C>T (homo.) | Levetiracetam, valproate, zonisamide |
| 4b | Leb | M | c.322C>T (homo.) | Levetiracetam |
| 5* | Rus | M | c.759delinsCATGCA | Perampanel, phenobarbital, valproate, brivaracetam |
| 6* | Ger | M | c.836G>T (homo.) | Brivaracetam, valproate, acetazolamide, perampanel, piracetam, clonazepam, topiramate, levetiracetam, zonisamide, rufinamide |
|  |  |  | ***EMP2B*** |  |
| 7* | Syr | F | c.385C>T (homo.) | Zonisamide, phenobarbital, valproate, perampanel, clobazam |
| 8 | Tur | M | c.436G>A (homo.) | Zonisamide, clonazepam, perampanel, valproate |
| 9 | Tur | F | c.436G>A (homo.) | Levetiracetam, oxcarbazepine, perampanel, valproate, gabapentin, lamotrigine, clobazam |
| 10* | Ger | F | c.[436G>A];[730delG] | Brivaracetam, valproate, acetazolamide, phenobarbital, levetiracetam, lamotrigine |
| 11* | Ira | M | c.583del (homo.) | Valproate, topiramate, clobazam, piracetam  *Peramapanel was not tolerated due to an increase of psychogenic seizures* |

Patients with novel variants are indicated by an asterisk (*). Patients 4 a and b are siblings. Abbreviations: Ger – German, Ira –Iraqi, Leb – Lebanese, Rus – Russian, Syr – Syrian, Tur – Turkish.
